# Endoscopic submucosal dissection of esophageal squamous cell carcinoma with superficial capillary changes and epithelialization after chemotherapy for pancreatic adenocarcinoma

**DOI:** 10.1055/a-2163-1658

**Published:** 2023-10-06

**Authors:** Alexandru Lupu, Sebastien Reynard, Jérôme Rivory, Pierre Lafeuille, Jean Grimaldi, Clara Yzet, Mathieu Pioche

**Affiliations:** 1 Gastroenterology and Endoscopy Unit, Edouard Herriot Hospital, Lyon, France; 2 Medical Center, Vindry-sur-Turdine, France

We present the case of a 79-year-old patient who was referred to us for endoscopic ultrasound evaluation of a cephalic pancreatic mass. He had a personal history of total laryngectomy for a T4 malignant tumor of the piriform sinus, with postoperative radiotherapy that ended 3 years previously. A phonatory cannula was in place from that moment on.

Initial endoscopic ultrasound identified a locally advanced pancreatic tumor invading the common hepatic artery, splenomesenteric confluence, and duodenum. During the same endoscopic examination, upon esophageal examination with narrow-band imaging, we identified a well-delineated superficial squamous cell lesion that had irregular and dilated intrapapillary capillary loops (IPCL) type V ( Video 1 ). Biopsies confirmed in situ squamous carcinoma.

**Video 1** White-light and narrow-band imaging of a superficial esophageal squamous cell carcinoma, before and after systemic chemotherapy for pancreatic adenocarcinoma.


The patient immediately started systemic chemotherapy with one cycle of FOLFOX (folinic acid, fluorouracil, and oxaliplatin) followed by six cycles of FOLFIRINOX (folinic acid, fluorouracil, irinotecan, oxaliplatin).

We present here white-light and magnification narrow-band imaging aspects of a superficial esophageal carcinoma with type V dilated and irregular IPCLs 1 2 3 , before and after chemotherapy for pancreatic adenocarcinoma. The esophageal lesion had suffered superficial alterations, with squamous epithelialization and a reduction in size, probably in response to the pancreatic systemic therapy, and showed de novo type II and III IPCLs, making it even more difficult to identify under endoscopic examination.

Despite systemic chemotherapy, the patient started presenting symptoms of digestive stenosis, which was related to duodenal invasion with consequent obstruction. We proposed and performed an endoscopic gastroentero anastomosis using a fully covered metallic stent (HOT Axios; Boston Scientific, Marlborough, Massachusetts, USA), as well as endoscopic submucosal dissection of the esophageal lesion ( Video 1 ) during the same general anesthesia procedure.

Histology showed complete endoscopic resection of a superficial, in situ squamous cell carcinoma, with negative lateral and vertical margins. He was discharged from our unit and started eating at Day 1 postintervention. Endoscopic re-evaluation 4 months later showed no residual lesion and no stenosis.

Endoscopy_UCTN_Code_TTT_1AO_2AG

## Notes

Endoscopy E-Videos

https://eref.thieme.de/e-videos
*E-Videos* is an open access online section of the journal *Endoscopy* , reporting on interesting cases and new techniques in gastroenterological endoscopy. All papers include a high-quality video and are published with a Creative Commons CC-BY license. Endoscopy E-Videos qualify for HINARI discounts and waivers and eligibility is automatically checked during the submission process. We grant 100% waivers to articles whose corresponding authors are based in Group A countries and 50% waivers to those who are based in Group B countries as classified by Research4Life (see: https://www.research4life.org/access/eligibility/ ). This section has its own submission website at https://mc.manuscriptcentral.com/e-videos
